# Investigation of PVA Matrix Hydrogel Loaded with *Centaurea cyanus* Extract for Wound Dressing Applications: Morphology, Drug Release, Antibacterial Efficiency, and In Vitro Evaluation

**DOI:** 10.3390/gels11040264

**Published:** 2025-04-02

**Authors:** Melis Abahuni Ucar, Enis Muhammet Gul, Deniz Uygunoz, Emek Moroydor Derun, Mehmet Burcin Piskin

**Affiliations:** 1 Department of Bioengineering, Yildiz Technical University, Istanbul 38000, Turkey; melisabahuni@hotmail.com (M.A.U.); mpiskin@yildiz.edu.tr (M.B.P.); 2 Department of Chemical Engineering, Yildiz Technical University, Istanbul 38000, Turkey; enisgul@yildiz.edu.tr (E.M.G.); duygunoz@yildiz.edu.tr (D.U.)

**Keywords:** *Centaurea cyanus*, hydrogel, wound dressing, polyvinyl alcohol

## Abstract

A polyvinyl alcohol (PVA) matrix hydrogel loaded with *Centaurea cyanus* extract was created for transdermal wound healing. Secondary metabolites, antibacterial properties, and the cytotoxicity of *C. cyanus* extract were investigated. The secondary metabolite profiles of the extract were determined by liquid chromatography–mass spectrometry (LC-MS) technique. It was determined that the extract has metabolites such as quinic acid, caffeoylquinic acid, kaempferol, etc., which contribute to the steps of wound healing. The extract had significant activity against *Staphylococcus aureus* when compared with ampicillin antibiotic and showed an inhibition zone of 16.9 mm ± 0.8, whereas ampicillin’s inhibition zone was 15.8 mm ± 0.8. The extract did not exhibit cytotoxic effects on 3T3-L1 (CL173) healthy skin fibroblasts, maintained cell viability for 72 h, and exhibited a 19% proliferative effect. Fourier-transform infrared spectroscopy, scanning electron microscopy, ultraviolet visible spectrophotometer, tensile strength analyses, in vitro release, and physicochemical tests were conducted. It was seen that the surfaces of the samples are smooth and homogeneous, patches had a significant amount of water absorption capacity, and 79% of the extract was released within the first 24 h of application; consequently, these results indicate that *C. cyanus* might be used in wound healing with its antibacterial, growth-promoting properties.

## 1. Introduction

Wound healing is a physiological, biochemical, and cellular process that involves all body systems that comprise the following four interacting steps: hemostasis, inflammation (1–4 days), proliferation (4–21 days), and tissue remodeling (21 days–2 years), respectively [1,2,3,4]. Wound treatment has always had an important role in the health field. Wounds that do not heal require long treatments and decrease the quality of life for the patient. Current methods, such as negative pressure therapy and surgery, are not always efficient treatments [5]; however, traditional dry wound dressings do not provide a moist environment for wound healing and do not have high antibacterial properties. An ideal wound dressing should be biocompatible and provide a moist environment to the wound. It should be easily removed from the tissue and must ensure adequate gas exchange and maintain an adequate wound surface temperature [6]. Transdermal skin patches are modern ideal wound dressings that meet these preferred features. Transdermal drug delivery systems, also known as transdermal patches, are dosage forms designed to deliver a therapeutically effective amount of active ingredient to the patient’s skin and, thus, to the bloodstream over time [7,8,9]. Plant extracts are preferred because they are natural and provide active ingredients [10].

Asteraceae is one of the plant families most used in wound treatment. Plants belonging to the Asteraceae family have ethnopharmacological importance in many societies, such as India, Turkey, Nigeria, Nepal, South Africa, Britain, and Ireland [11]. Due to this importance, various plants from this family have been studied for their pharmacological activities, such as anti-inflammatory, antimicrobial, and healing activities [12,13,14]. The therapeutic potential of some species, such as *Calendula officinalis* L., *Achillea millefolium* L., and *Neurolaena lobata* (L.), in wound care has been reported in the literature [15,16]. It has been stated that their activities are related to their ability to promote the proliferation of keratinocytes and, thus, the remodeling of the extracellular matrix [17,18]. Although there are various studies in the literature on other species belonging to the Asteraceae family, there is limited information about *Centaurea cyanus*. The following information is generally related to the determination of bioactive components, taxonomic classification, and antioxidant, antimicrobial, and anti-inflammatory properties of *C. cyanus* [19,20,21,22,23,24,25,26]. *C. cyanus* has traditionally been used in areas such as eye care and wound healing. In one study, the upper and lower parts of the capitulum of *C. cyanus* were examined and compared in terms of their chemical composition and bioactive properties. It has been observed that the flower parts are rich in tocopherol, citric acid, and apigenin derivatives, while the lower parts are rich in quinic acid and syringic acid. As a result, the lower part of the plant has been shown to exhibit antioxidant and antibacterial properties that can be used in different food or pharmaceutical formulations [27].

To the best of our knowledge, the published literature did not provide information on a therapeutic transdermal patch containing *C. cyanus* extract; therefore, the aim of the present study was to design a polyvinyl alcohol (PVA) matrix transdermal patch involving *C. cyanus* extract. The developed transdermal patches and the plant extract used were subjected to a series of analyses comprising liquid chromatography mass spectrometry (LC-MS), antibacterial tests, 3-(4,5-dimethylthiazol-2-yl)-2,5 diphenyl tetrazolium bromide (MTT), Fourier-transform infrared spectroscopy (FTIR), scanning electron microscopy (SEM), ultraviolet visible spectrophotometer (UV-Vis), tensile strength tests, and several of other physicochemical tests.

## 2. Results and Discussion

### 2.1. LC-MS Results

**Figure 1:**
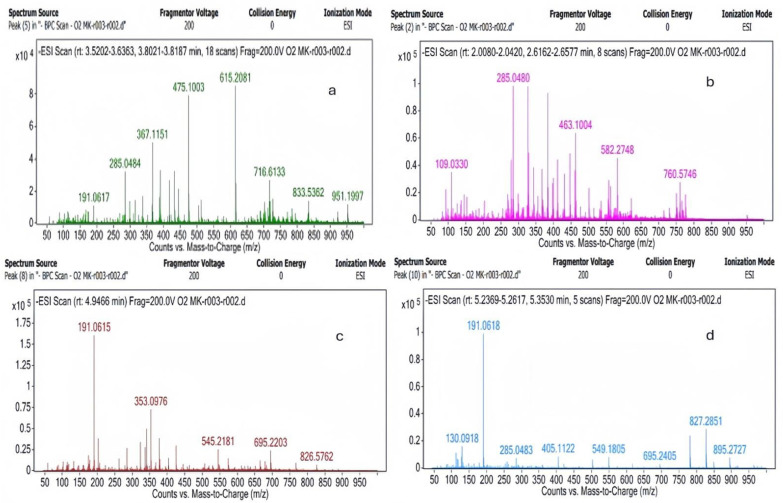
(**a**–**d**) The liquid chromatography mass spectrometry results obtained for the components of *Centaurea Cyanus*.

It was observed that the effectiveness of the hypericin and hyperforin components was minimal (Table 1). It was concluded that the effectiveness of *C. cyanus* extract was derived from the quinic acid, kaempferol, quercetin-O-(O-galloyl)-hexoside, caffeoylquinic acid, hyperoside, and adhyperforin components observed at *m*/*z* = 191, 285, 615, 353, 463, and 549, respectively, when compared with that in the literature [28,29,30,31,32,33,34] (Figure 1).

These metabolites have the potential to positively affect the phases of wound healing. The coagulating property of quinic acid contributes to the hemostasis phase. Kaempferol exhibits high antioxidant properties, interacts with reactive oxygen species (ROS) that cause tissue damage, and reduces the ROS rate. In a study conducted using rats, it has been reported that the quercetin component contributes to dermal wound healing by increasing growth factors and also forms a collagen matrix; therefore, because of the quercetin component in *C. cyanus* extract, it would develop new tissue consisting of collagen and the extracellular matrix, as a contribution to the formation of new tissue in the proliferative phase. Because caffeoylquinic acid has anti-inflammatory and antihyperlipidemic properties, it is predicted to have a positive effect during wound healing by preventing inflammation [35,36,37,38].

### 2.2. MTT Results

**Figure 2:**
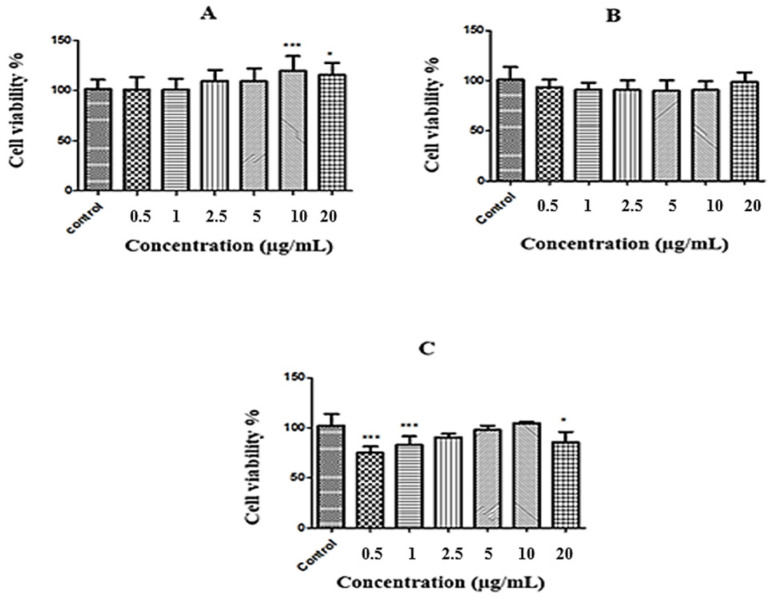
(**A**) Effects of the sample on NIH-3T3 cell viability after 24 h of exposure, (**B**) effects of the sample on NIH-3T3 cell viability after 48 h of exposure, and (**C**) effects of the sample on NIH-3T3 cell viability after 72 h of exposure. Notes: Data are mean ± standard deviation, * *p* < 0.05, and *** *p* < 0.001 versus the control group.

According to the results of the cytotoxicity experiment performed to evaluate the plant extract interaction in skin tissue, the IC_50_ value for the analyzed sample was determined to be >20 µg/mL. The results of evaluating the sample for three different time periods and six different concentrations are shown separately in Figure 2.

According to MTT analysis results, *C. cyanus* extract did not cause 50% cell viability inhibition at the studied concentrations and was not considered toxic within the studied concentration range. Compared with the control group, a 19% proliferative effect was observed at a concentration of 10 µg/mL and 15% at a concentration of 20 µg/mL. This shows that *C. cyanus* extract may have positive effects on the wound healing process.

### 2.3. Antibacterial Activity Results

**Figure 3:**
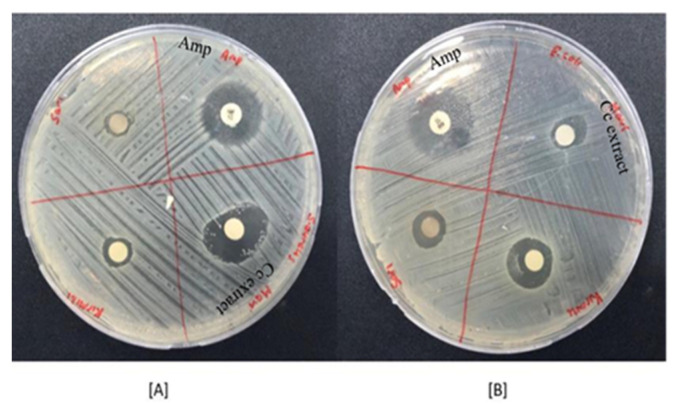
Interaction of *Centaurea cyanus* extract *on Staphylococcus aureus* (**A**) and *Escherichia coli* (**B**) cultures.

Figure 3 shows the inhibition zone formations of *C. cyanus* extract on *E. coli* and *S. aureus* cultures, and the diameters of the inhibition zones were measured. It was shown in Table 2.

The results showed that the extract had antibacterial activity on both *E. coli* and *S. aureus*. It was also observed that the extract had more antibacterial activity on *S. aureus* than on the ampicillin, which was the control group. This antibacterial activity resulted from the antimicrobial and antibacterial properties of the quinic acid, kaempferol, quercetin, hyperforin, and hypericin metabolites of *C. cyanus* extract [39,40,41].

### 2.4. Characterization of Herbal Transdermal Patch

#### 2.4.1. SEM

**Figure 4:**
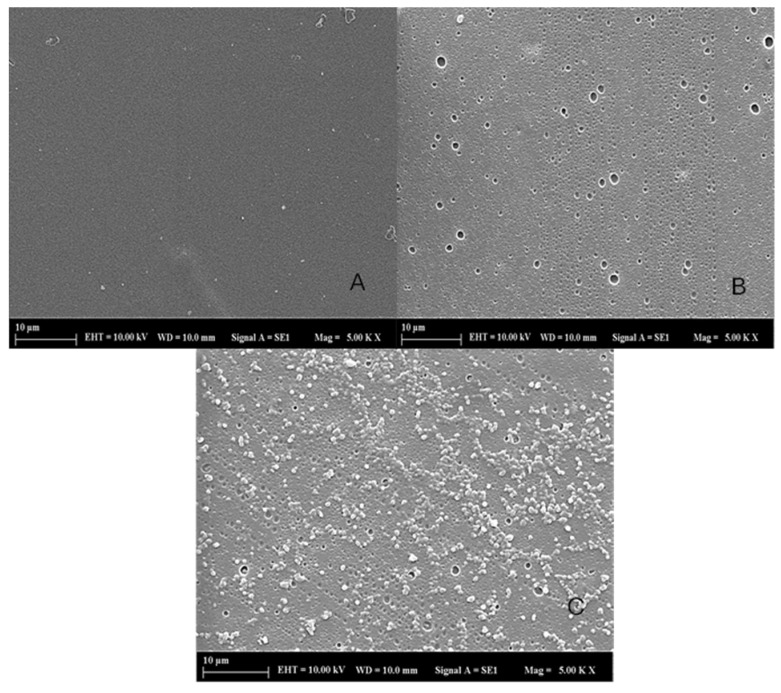
Scanning electron microscopy of (**A**) Pure polyvinyl alcohol transdermal patch image (5 KX), (**B**) herbal transdermal patch image (5 KX), and (**C**) post-release image of herbal transdermal patch after 24 h of dissolution (5 KX).

It was observed that the surfaces of the samples were smooth and homogeneous, and shown in Figure 4. It was observed that the herbal formulation exhibited a porous structure, which contributes to tissue healing by ensuring oxygen permeability, absorbing wound exudate, creating entry and exit pathways for water and active substances, and providing a moist environment to the wound [42].

#### 2.4.2. FTIR

**Figure 5:**
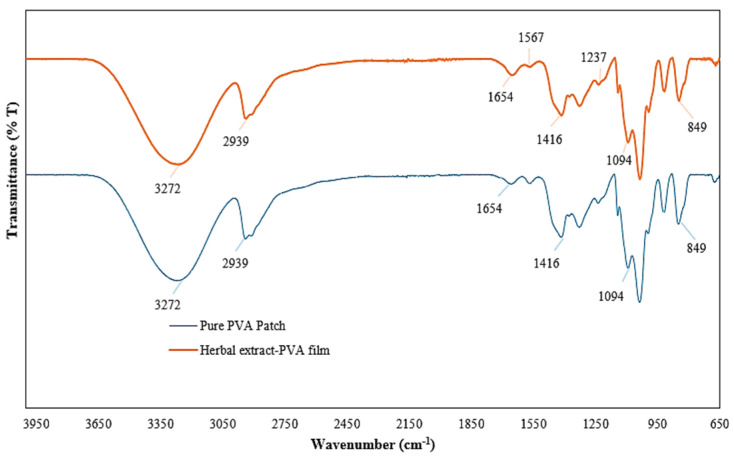
Fourier-transform infrared spectroscopy results of pure polyvinyl alcohol transdermal patch and herbal transdermal patch.

FTIR spectra of the pure and herbal transdermal patches were determined using FTIR and shown in Figure 5. The pure PVA transdermal patch spectrum exhibited characteristic peaks at 3272, 2939, 1654, 1416, 1094, and 849 cm^−1^, which are related to O-H, C-H, C=O, C-H, C-O, and C-C stretching [43,44]. After adding *C. cyanus* extract, the characteristic peaks of PVA were still observed, which meant that the extract did not affect the main molecular structure of PVA. In addition, with the addition of the extract, new peaks were observed at 1567 and 1237 cm^−1^ wavelengths, which correspond to C=C and C-C stretching vibrations in the aromatic ring of flavonoids [45,46,47]. In general, it was observed by FTIR analysis that the extract was successfully incorporated into the PVA hydrogel.

#### 2.4.3. Tensile Strength Test

**Figure 6:**
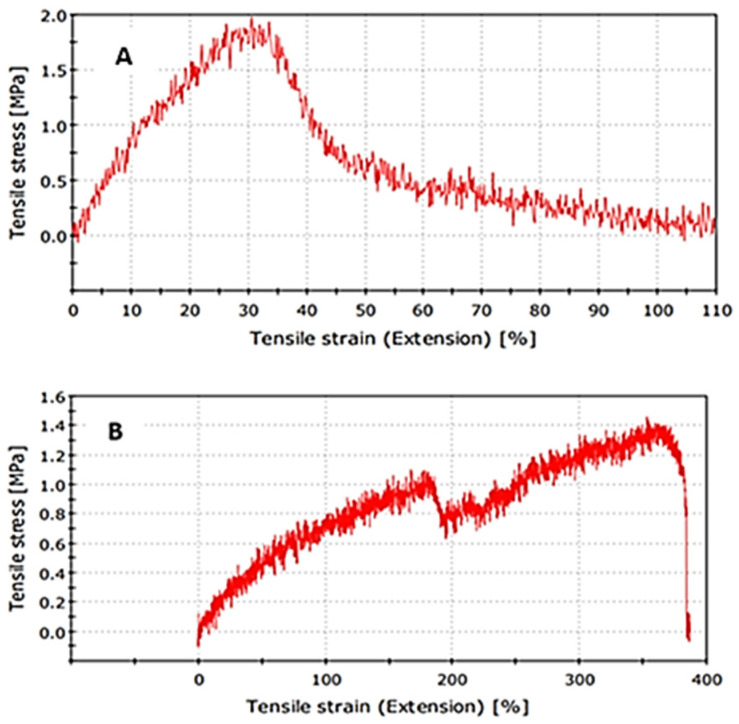
Tensile strength results of (**A**) pure polyvinyl alcohol transdermal patch and (**B**) herbal transdermal patch.

The results of the tensile strength test are shown in Figure 6. The breaking tensile strength of the pure PVA transdermal patch was 1.87 MPa, and that of the herbal transdermal patch was 1.37 MPa. This situation occurs as a result of the decrease in the polymer solution ratio in the formulation and the increase in porosity with the addition of the plant extract.

#### 2.4.4. Determination of Transdermal Patch Thickness

The thicknesses of synthesized samples are shown in Table 3. The average thickness of the pure PVA transdermal patch was 0.147 ± 0.03 mm, and that of the herbal transdermal patch was 0.16 ± 0.03 mm. It was clearly observed that herbal patch thickness was greater than that of the pure patch.

#### 2.4.5. Weight Uniformity

The weights of the synthesized samples are shown in Table 3. It was observed that the weights of both the pure PVA transdermal patch and the herbal patch were uniform throughout the transdermal patch.

#### 2.4.6. Moisture Content Percentage

The moisture contents of the synthesized samples are shown in Table 3. The percent moisture content of transdermal films may vary depending on the amount of polymer solution in their formulation. In addition, the plant extracts they contain and their amounts are also factors that affect the moisture content in the formulation. In this context, the data obtained were found to be compatible with those in the literature [48,49].

#### 2.4.7. Percentage of Dehumidification

The percentages of dehumidification of the synthesized samples are shown in Table 3. It was determined that the moisture absorption percentage of the formulation containing the plant extract was relatively higher than that of the pure patch. The data obtained were compatible with those in the literature [50].

#### 2.4.8. Percent Swelling Rate

**Figure 7:**
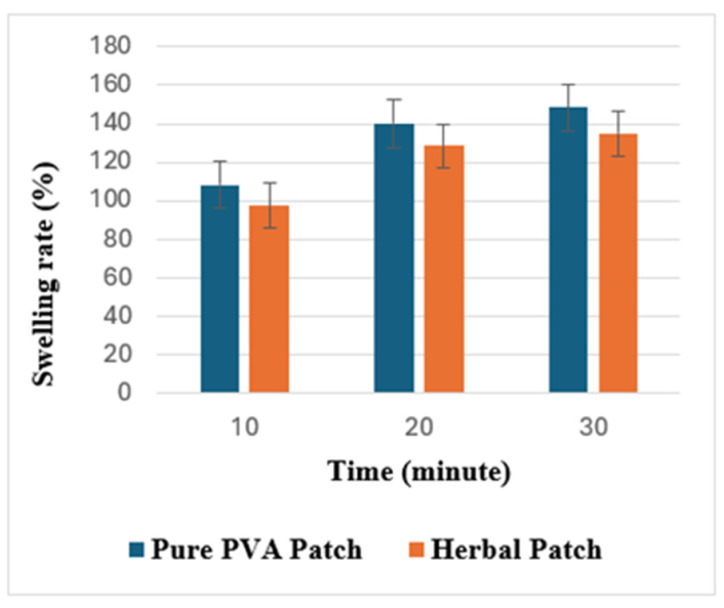
Percent swelling rate test. Notes: *n* = 3, mean ± SD.

The percent swelling ratio test findings of the optimum synthesized samples are shown in Figure 7. For skin renewal and wound healing to occur, the wound area must be moist, and the wound dressing must absorb wound fluid and exudate; therefore, the swelling feature is important. Pure PVA transdermal patches exhibited considerable water absorption capacity between 108.2 and 148.5%, and the herbal transdermal patches exhibited water absorption capacity between 97.4 and 135.2%, which suggested an important potential for these patches in applications that require substantial moisture retention. It was observed that the resistance of transdermal films to swelling increased in the herbal formulation. This positive result was created from the interaction between the polymer and plant extract in the formulation. The findings obtained are close to those in the literature.

#### 2.4.9. Determination of Surface pH

The pH of each synthesized sample is shown in Table 3. It was observed that the average pH of both samples was comparable and compatible with skin pH.

#### 2.4.10. In Vitro Release Profile

**Figure 8:**
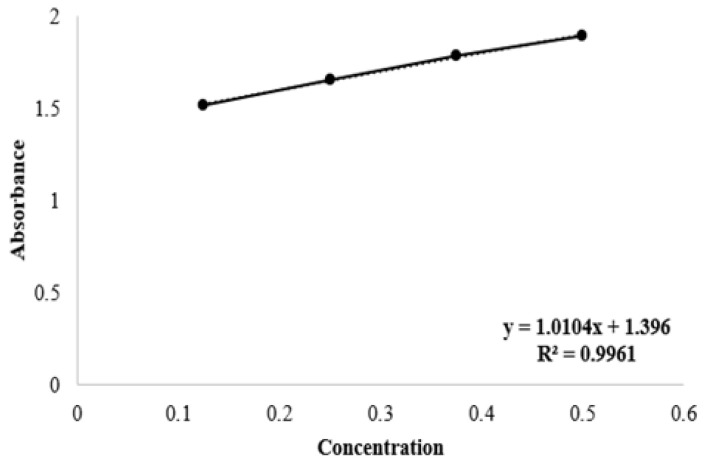
The standard calibration curve of the extract.

The standard curve of the extract was obtained using different concentrations of *C. cyanus* and the UV-Vis spectrophotometer, and shown in Figure 8.

**Figure 9:**
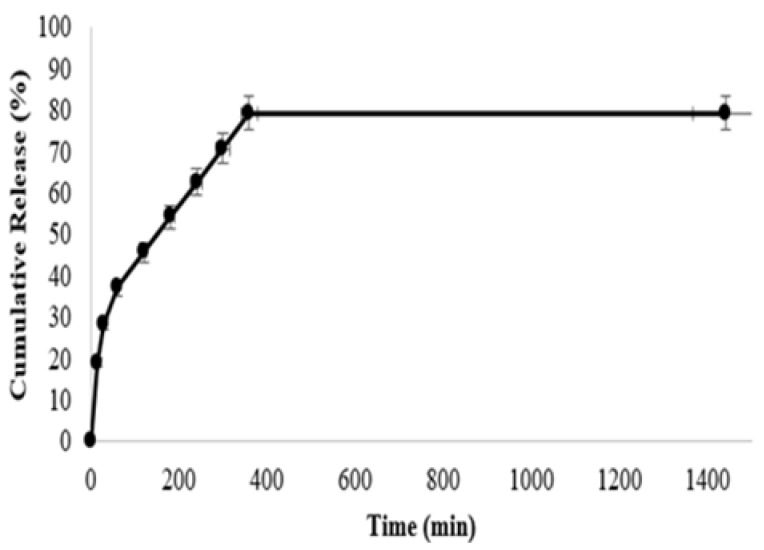
The cumulative release profile of the extract.

The cumulative release amount of extract is shown in Figure 9. A burst release at first and then a linear and balanced release graph was observed. Extract release within the first 60 min was 19.17%, 70.83% at 360 min, and at the 24th hour, it was 79%. The 70% release that occurred within the first 6 h plays an important role in preventing bacterial growth and reducing the effect of ROS in the initial phase of wound healing [51,52].

## 3. Conclusions

In the present study, a PVA matrix transdermal wound dressing containing *C. cyanus* extract was successfully fabricated and evaluated for its wound-care potential. It was observed that the extract has secondary metabolites, such as quinic acid, caffeoylquinic acid, and kaempferol, which contributed to the steps in wound healing. In addition, it was observed that the extract had significant activity against *S. aureus* as well as ampicillin antibiotic, which has an inhibition zone of 15.8 mm ± 0.8 and an inhibition zone of 16.9 mm ± 0.8. The extract did not exhibit cytotoxic effects on 3T3-L1 (CL173) healthy skin fibroblasts, did not cause 50% cell viability inhibition at the studied concentrations, maintained cell viability for 72 h, and exhibited 19% proliferative effect at a concentration of 10 µg/mL and 15% at a concentration of 20 µg/mL. According to FTIR results, the PVA functional groups were confirmed for both the pure and extract-loaded samples. SEM analysis showed that the extract-loaded formulation had a porous structure. The tensile test revealed the durability of the synthesized sample. Physicochemical test results showed compatibility with that in the literature. In addition, it was observed that the patches had a significant amount of water absorption capacity. Moreover, 79% of the extract was released within the first 24 h, and 70% release occurred within the first 6 h, which plays an important role in preventing bacterial growth and reducing the effect of ROS in the initial phase of wound healing. In light of this information, it was suggested that the material was successfully synthesized and that *C. cyanus* might be used in wound healing because of its antibacterial, growth-promoting properties, and would be a new alternative to today’s wound treatments. In conclusion, the wound healing potential of PVA matrix hydrogel loaded with *Centaurea cyanus* extract was demonstrated in this study. These findings underline the need for further research and development in this area.

## 4. Materials and Methods

### 4.1. Materials

*Centaurea cyanus* plants were obtained from a local sapling market in Istanbul, Turkey. The microorganism strains of *Staphylococcus aureus* (ATCC 25923) and *Escherichia coli* (ATCC 25922) were used as test bacteria. Nutrient agar, nutrient broth, and absolute ethanol (C_2_H_5_OH) with a purity of >99.8% and molecular weight of 46 g/mol were purchased from Merck (Darmstadt, Germany). PVA with a purity of 99%+ hydrolized and molecular weight of Mw 85,000–124,000 was obtained from Sigma-Aldrich (Burlington, MA, USA). Glycerine with a purity of 99.5% and molecular weight of 92.10 g/mol was supplied from Tekkim (Bursa, Istanbul). Phosphate buffer solution (PBS) at pH 7.4 was retrieved from Nacalai Tesque (Kyoto, Japan). Distilled water was used throughout all experiments.

### 4.2. Synthesis Procedure

#### 4.2.1. Plant Extract 

The dried plants were ground using a grinding device. Soxhlet extraction using a Velp Scientifica Ser 148, Italy, was used as the preferred extraction method. Ethanol was used as the extraction solvent. The device consisted of the following three steps: (1) 60 min immersion, (2) 60 min washing, and (3) 10 min recovery at 210 °C. In the first step, the plants were soaked in the boiling extraction solvent. In the second step, the plants were removed from the solvent and washed with hot ethanol. In the final step, a large proportion of the solvent was recovered by distillation. The resulting plant extract was stored in amber glass bottles at room temperature in a desiccator.

#### 4.2.2. Fabrication of Herbal Transdermal Patch

The transdermal patches were prepared using the solvent-casting method. Pure PVA solution was prepared using the appropriate temperature and 750 rpm stirring speed. Homogenization was achieved by taking a certain amount of PVA solution according to the formulation and adding the appropriate amount of plasticizer (glycerin). Then, 1.5 mL of the plant extract was added to the solution, mixed (30 min at 750 rpm), and set into an ultrasonic bath. The prepared samples were placed in Petri dishes and left to dry in an oven.

### 4.3. Characterization

#### 4.3.1. LC-MS Method

LC-MS was used to determine the hypericin and hyperforin, phytochemical components in the plant extracts that are the most common metabolites in *Asteraceae*, according to the literature [53]. The *C. cyanus* extract was analyzed using an Agilent 6530 LC MS-TOF system, Santa Clara, CA, USA. Standard samples of hypericin and hyperforin were used, and their quantitative determination was performed using tandem mass spectrometry (MS/MS) operated in negative mode. The quantification was based on the quantity and purity values indicated on the standard sample certificates. The amount of hypericin in the analytical standard sample was 10 mg with 88.90% purity. The amount of hyperforin in the analytical standard was 250 µg with >85% purity. The hypericin and hyperforin analytes were determined using a *C. cyanus* (02-MK) sample. An ACE 3 µm Sil column was used, and the mobile phases comprised phase A (100 mM ammonium formate in distilled water [pH 3]/acetonitrile [19:1 *v*/*v*]) and Phase B (100 mM ammonium formate in distilled water [pH 3]/acetonitrile [1:19 *v*/*v*]). The column temperature was 40 °C.

#### 4.3.2. MTT Test

MTT analysis was conducted to evaluate the cytotoxicity of the desired extract. 3T3-L1 (CL173) healthy skin fibroblast systems were used. The live broadcast rate was run using quantitative colorimetry with a spectrophotometer with a microplate reader. After the cells were incubated in an oven with 5% CO_2_ at 37 °C for 24 h, six different concentrations of the samples were prepared with the new medium (final distribution 20–0.5 µg/mL) and repeated in at least two technical and three biological replicates. It was applied and incubated for 24, 48, and 72 h. The percent viability fractions of the concentration were calculated based on the control group. The one-half maximal inhibitory concentration (IC_50_) values were calculated by probit analysis using the GraphPad Prism 5 program (Windows 5.04). One-way analysis of variance and Tukey’s post hoc test were used for statistical analyses.

#### 4.3.3. Antibacterial Activity

The antibacterial activity of *C. cyanus* extract was determined using the disc diffusion method. *E. coli* ATCC 25922, a gram-negative bacterium, and *S. aureus* ATCC 25923, a gram-positive bacterium, were used for the study, along with an ampicillin antibiotic disc as a control.

#### 4.3.4. Characterization of Herbal Transdermal Patch

##### SEM

The surface morphology of the patches was analyzed using SEM, and a Zeiss EVO LS 10, Oberkochen, Germany, at 5.00 kX magnification with an accelerating voltage of 10 kV was used for analyses.

##### FTIR

FTIR analysis was conducted to gather information about the structure of the raw materials used in the formulation and forming of the developed transdermal patches. The analysis was conducted using the attenuated total reflectance technique on the Perkin Elmer-Spectrum 100 device, Waltham, MA, USA, at a wave range of 4000–650 cm^−1^.

##### Tensile Strength Test

The Instron 5982 Electromechanical Universal Testing Machine, Wycombe, UK, was used for the analysis. The sample reference length was determined to be 10 mm, and the cross-head speed was determined to be 10 mm/min.

##### Determination of Film Thickness

The thickness of the developed transdermal films was measured using a digital micrometer by taking samples from three different points on the film. The average thickness was then determined along with the standard deviation [54,55].

##### Weight Uniformity

Circular sections with a diameter of 2 cm were taken from three different regions of the prepared patches and weighed on an analytical balance. The average weight was then determined along with the standard deviation [56].

##### Moisture Content Percentage

The moisture content of the produced transdermal patches was determined using the Radwag brand moisture analyzer, Poland. The moisture content of the transdermal films was determined by the initial weight of the sample before drying, which was calculated as the percentage difference between the sample weights before and after drying. The average percent moisture content was calculated for three replicates along with the standard deviation [56].

##### Percentage of Dehumidification

The prepared transdermal patch formulations were weighed on an analytical balance and kept in a desiccator containing saturated potassium chloride solution for 24 h to ensure 84% Rhesus factor, after which the transdermal formulations were weighed again, and the moisture absorption percentages were determined using Equation 1 as follows [56]:

Moisture absorption percentage (%) = (final weight − initial weight)/initial weight × 100(1)

##### Percent Swelling Rate

Circular sections of 2 cm in diameter were cut from three different parts of the produced patches and weighed on an analytical balance, after which the samples were placed in Petri dishes containing distilled water and subjected to swelling tests at 10 min intervals for a total of 30 min. The average swelling ratio was determined for three different samples [56].

##### Determination of Surface pH

Circular sections with a diameter of 2 cm were taken from the developed transdermal films, and the pH was measured using a digital pH meter. All tests were repeated three times, and the average was calculated along with the standard deviation.

##### In Vitro Release Profile

For the in vitro release study, a Franz diffusion cell was used with cellulose acetate membrane, using a PBS solution with a pH of 7.4 as the release medium and a temperature of 37 ± 1 °C [57]. Ultraviolet measurements were made by taking samples at specific time intervals (0, 15, and 30 min and 1, 2, 3, 4, 5, 6, and 24 h), and the concentrations of the samples was calculated using a calibration curve. The calibration curve was created by determining the values of the extract at different concentrations corresponding to 206 nm, which is the wavelength at the maximum absorption point.

## Figures and Tables

**Table 1:** Determination of hypericin and hyperforin of *Centaurea cyanus* extract.

Plant	Hypericin Amount (ng mL^−1^)	Hyperforin (ng mL^−1^)
*Centaurea cyanus*	12.5 ± 5.2 *	43.2 ± 0.7 *

* *n* = 3, mean ± SD.

**Table 2:** Inhibition zone diameter created by *Centaurea cyanus* extract on *Escherichia coli* and *Staphylococcus aureus*.

Sample	*E. coli* (mm)	*S. aureus* (mm)
*Centaurea cyanus*	8.2 ± 0.5 *	16.9 ± 0.8 *
*Ampicillin*	17.1 ± 0.6 *	15.8 ± 0.8 *

* *n* = 3, mean ± SD.

**Table 3:** Physicochemical test results.

Parameters	*Pure PVA Patch*	*Herbal Patch*
Patch thickness (mm)	0.147 ± 0.03 *	0.16 ± 0.03 *
Weight uniformity (g)	0.0555 ± 0.01 *	0.0775 ± 0.01 *
Moisture content percentage (%)	8.33 *	10.87 *
Humidification percentage (%)	2.73 *	3.91 *
Surface pH	6.87 ± 0.03 *	6.92 ± 0.03 *

* *n* = 3, mean ± SD.

## Data Availability

The original contributions of this study are included in the article. Further inquiries can be directed to the corresponding author.
